# Approach to different thrombolysis techniques and timing of thrombolysis in the management of portal vein thrombosis in cirrhotic patients

**DOI:** 10.2478/jtim-2023-0113

**Published:** 2023-09-02

**Authors:** Massimo Primignani, Giulia Tosetti, Anna Maria Ierardi

**Affiliations:** Fondazione IRCCS Ca' Granda Ospedale Maggiore Policlinico, Milan 20122, Italy; Department of Radiology Fondazione IRCCS Ca' Granda Ospedale Maggiore Policlinico di Milano, Milan 20122, Italy

**Keywords:** thrombolysis, portal vein thrombosis, cirrhosis

## Abstract

Thrombolysis is not currently recommended in cirrhotic patients with acute portal vein thrombosis (PVT) in most guidelines, because of the exceedingly limited data and the perceived high risk of bleeding adverse events. However, in the few studies including patients with cirrhosis, the rate of success was high and that of adverse events was similar in patients with or without cirrhosis. Hence, thrombolysis might be a rescue therapeutic option in patients with cirrhosis and acute, symptomatic thrombosis of the portal venous system, unresponsive to anticoagulation, provided a suitable timing is kept, less than 30 days and, if possible, less than 14 days from the acute onset of portal vein thrombosis. In this review perspective article, I discuss the several potential approaches of thrombolysis, either local or systemic, alone or combined with mechanical procedures for thrombus removal, or as a complement to Transjugular Intrahepatic Portosystemic Shunt placement, with a focus on the more suitable timing of thrombolysis. However, the very limited available data preclude from performing firm recommendations, and decision to carry out thrombolysis must take into account both the occurrence of major contraindications and the current critical clinical setting. In the next future, large high-quality multicentre studies will hopefully be able to settle more firm indications and preferable techniques.

## Introduction

Portal vein thrombosis (PVT) occurs in 7% to 17% of patients with cirrhosis, with the higher prevalence in the advanced stages of the disease.^[1]^ In most cases, PVT is partial and asymptomatic, incidentally detected at periodic imaging surveillance.^[2]^ The impact of such kind of PVT on the natural history of cirrhosis is controversial ^[3, 4, 5, 6]^ and, consequently, its management is variable and assessed on a case-by-case basis, mainly taking into account its tendency to progress on short-term follow-up (1–3 months), the involvement of the superior mesenteric vein and the patient’s candidacy to liver transplantation. Taking into account the above variables, careful watching or anticoagulation is currently proposed for partial, asymptomatic PVT in patients with cirrhosis, whereas thrombolysis has no role in such clinical setting.^[7]^

Conversely, in a minority of patients with cirrhosis, PVT may have an abrupt onset with ominous sequelae, such as mesenteric ischemia or infarction or variceal bleeding, and a high mortality rate.^[8,9]^

Whereas anticoagulation is the first line treatment also in acute, symptomatic PVT,^[10,11]^ further, more aggressive treatments may be early applied, particularly in the case of lack of resolution/aggravation of symptoms or progression of thrombosis, with an impending risk of mesenteric infarction.^[11]^ In such cases, systemic or local thrombolysis, percutaneous portal vein recanalization, and transjugular intrahepatic portosystemic shunt (TIPS), alone, in combination, or in sequence, can be considered.

In this review perspective article, I discuss the several potential approaches of thrombolytic therapy, with a focus on the proper timing of thrombolysis in cirrhotic patients with acute PVT. Although such treatments have been increasingly and often successfully performed in recent years,^[12, 13, 14, 15, 16, 17, 18, 19, 20, 21, 22, 23, 24, 25]^ it must be acknowledged that data of thrombolytic treatment in patients with acute PVT, and even more in acute PVT in cirrhosis patients, come from anecdotal case reports or few small case series, and not from relevant cohort studies or controlled clinical studies. Of note, most of such studies include patients with non-cirrhotic PVT and very few include patients with cirrhosis and acute PVT.^[12, 13, 14, 15, 16, 17, 18, 19, 20, 21, 22, 23, 24, 25]^ (Table 1) However, even in such very few studies, thrombolytic treatment appeared often effective, with adverse events occurring at variable rates in different series, but apparently similar in patients with or without cirrhosis. Nonetheless, data on safety in patients with cirrhosis remain very scarce. Overall, the thrombolytic treatments herein discussed should be considered as possible therapeutic options, still not supported by clinical guidelines, in front of a life-threatening, dramatic clinical condition. Either a local or a systemic approach may be accomplished, though the former is the most frequently attempted. It must be underlined that both types of procedures carry a high bleeding risk, and are only justified as rescue measures in front of failure of anticoagulation as the first line therapeutic approach.^[26]^ Drugs used for thrombolysis include streptokinase, urokinase or recombinant tissue plasminogen activator (rTPA).

**Table 1:** Current evidence for thrombolysis for portal vein thrombosis in cirrhosis

**References**	**Technical approach**	**Number of patients**	**PVT resolution**	**Serious adverse events**
De Santis^[12]^, 2010	systemic	9	4 complete	none
			4 partial	
			1 no change	
Malkowski^[13]^, 2003	systemic	28	10 complete	none
			13 partial	
			5 no change	
Jiang^[15]^, 2017	local, *via* SMA	20		none
Blum^[16]^, 1995	TIPS plus low-dose thrombolysis	7		none
Rosenqvist^[17]^, 2016	TIPS plus thrombolysis	11		none
Rossi^[19]^, 2004	Thrombectomy plus thrombolysis	3	complete	none
Rusu^[24]^, 2020	local, for TIPS acute occlusion	1	complete	none
Gabler^[25]^, 1997	combined local and systemic	1	complete	none

PVT: portal vein trombosis; TIPS: transjugular intrahepatic portosystemic shunt.

Thrombolysis, either local or systemic, should not be carried out in the presence of absolute contraindications,which include central nervous system tumours, recent haemorrhagic stroke, recent or uncontrolled gastrointestinal bleeding and severe, uncontrolled hypertension.^[27]^ Relative contraindications include pregnancy, remote history of GI bleeding, and recent major surgery.^[27]^ Moreover, the indications of thrombolysis should be limited to recent acute PVT (*i.e.* less than 30 days, possibly less than 14 days), not to chronic PVT (*i.e.* portal cavernoma or fibrous cord).^[11]^

Many techniques are available, such as TIPS with mechanical aspiration thrombectomy and/or direct thrombolysis, percutaneous transhepatic thrombectomy or thrombolysis, indirect thrombolysis *via* the superior mesenteric artery and thrombolysis *via* a surgically placed superior mesenteric vein catheter.

TIPS is recommended in patients with thrombosis of the portal vein trunk without recanalization on anticoagulation, especially in patients listed for liver transplantation for restoring portal patency,^[9]^ improving portal flow and decompressing portal hypertension. The important role of TIPS in the management of acute PVT in cirrhosis patients is discussed in other places. Herein I only discuss the possible added role of endovascular interventional techniques, either mechanical or pharmacological, to restore hepatopetal flow in patients with cirrhosis and PVT treated with TIPS because of contraindications or failure of anticoagulation treatment.

## Systemic thrombolysis

The experience with systemic thrombolysis in patients with cirrhosis and acute PVT is exceedingly limited. Systemic thrombolysis requires intravenous infusion of a thrombolytic drug, streptokinase, urokinase, or recombinant tissue plasminogen activator (rTPA), usually in conjunction with Low Molecular Weight Heparin (LMWH). A small pilot study^[12]^ on nine cirrhotic patients with recent PVT (< 30 days) undergoing continuous rTPA intravenous infusion (0.25mg‧kg^−1^‧d^−1^) plus subcutaneous LMWH up to 7 days achieved positive results, with complete PVT resolution in four cases and partial regression in four, without clinically significant adverse events.

A retrospective study^[13]^ of 33 patients with acute cirrhotic- or non-cirrhotic PVT, mainly treated with rTPA demonstrated complete recanalization in 10 patients with PVT not exceeding 14 days, partial, but sufficient recanalization in 13 patients with longer duration of PVT but less than 30 days, and no resolution in 5 patients with PVT lasting longer than 30 days. Of note, conservative treatment was unsuccessful in five cases and all of them died from esophageal variceal bleeding and liver failure. In this study, although the inherent biases of a retrospective study, early thrombolytic treatment of acute PVT was the only way to improve, or even restore the hepatopetal flow.

## Local thrombolysis

Local thrombolysis can be performed directly, *via* the percutaneous transhepatic, transjugular, or transmesenteric route, or indirectly, *via* the superior mesenteric artery, through a radial or femoral artery access.^[14,15]^ The latter approach was recently applied on 40 cirrhosis patients with acute PVT,^[15]^ randomized to transcatheter selective superior mesenteric artery (SMA) urokinase infusion (500,000 IU bolus, 250,000– 500,000 IU, depending on the patient’s weight, twice daily, for a total of 15,000 IU‧kg^−1^‧d^−1^) *vs.* TIPS. Thrombolysis time depended on the improvement of the patient’s clinical symptoms, a decrease in D-dimer (constantly monitored), and imaging data, performed at intervals of 72 hours.^[15]^ Both procedures required also anticoagulation, subcutaneous heparin (2000 IU, twice daily in the thrombolysis group or lLMWH 6000 IU, three time daily for one week in the TIPS group), and warfarin thereafter for three to six months.

Both treatments similarly reduced acute PVT, without difference between them and without serious adverse events. In detail, symptoms improved in all patients within 48 hours, PVT improved in 17 (85%) patients in the SMA group and 14 (70%) patients in the TIPS group, whereas superior mesenteric vein thrombosis and splenic vein thrombosis were reduced only in the SMA group, suggesting such treatment as the most suitable for treating recent mesenteric vein thrombosis. Of note, the occurrence of hepatic encephalopathy was significantly higher in the TIPS group.^[15]^

## Combined treatments

### TIPS combined with low-dose thrombolysis and/or mechanical thrombectomy

TIPS combined with low-dose thrombolysis was successfully attempted in seven patients with cirrhosis and complete, non-cavernomatous portal vein occlusion and recurrent variceal bleeding, with restored portal blood flow and without bleeding complications.^[16]^

Another study^[17]^ achieved recanalization in eleven cirrhotic patients with PVT and variceal bleeding or refractory ascites treated with TIPS combined with local thrombolysis, hence concluding that TIPS combined with thrombolysis should be considered in patients with extensive PVT and bowel ischemia, though the results were suboptimal, with re-intervention needed in five patients and recurrences of variceal bleeding in three.

Besides thrombolysis, further options for recanalizing the occluded portal vein are available, such as devices for mechanical suction of thrombus that may achieve a quicker removal of the thrombus, thus limiting or avoiding the protracted infusion of a thrombolytic agent and its related bleeding risks, particularly indicated in patients with contraindications to thrombolysis. In some cases, mechanical thrombectomy may require balloon angioplasty with/without stent placement, mainly for chronic, organized thrombosis. Mechanical removal of thrombus may be associated with a higher risk of trauma of the vascular wall and the ensuing risk of thrombosis recurrence.

Actually, in a meta-analysis^[18]^ including 399 patients, mostly with cirrhosis, assessing the effect of TIPS on portal vein recanalization, additional catheter-directed thrombolysis was associated with more complications than TIPS alone or TIPS plus mechanical thrombectomy (17.6% *vs.* 3.3%).

### Mechanical thrombectomy plus thrombolysis in transplanted patients with portal vein thrombosis

Combined mechanical and pharmacologic thrombolysis for portal vein thrombosis in liver-graft recipients and in candidates for liver transplantation was successfully performed in three patients, thus saving the liver graft or allowing orthotopic liver transplantation.^[19,20]^ The thrombectomy device was used through the percutaneous transhepatic access and the best results were achieved by performing mechanical thrombectomy before urokinase infusion, hence allowing a limited dosage of urokinase to achieve recanalization and preventing bleeding adverse events.^[19]^

### Thrombolysis for acute TIPS occlusion

Acute TIPS occlusion, due to biliary leakage or stent malposition occurs in 10%–15% of patients undergoing TIPS with bare stents^[21]^ and in 4%–5% of the patients undergoing TIPS with a covered stent.^[22,23]^ rTPA local infusion^[24]^ or combined local (10 mg r-TPA) and systemic (100 mg r-tPA) thrombolysis^[25]^ successfully recanalized PVT after TIPS.

## Conclusive remarks

Thrombolysis in cirrhosis patients with acute PVT is reported in small case series or case reports, mainly as rescue therapy in case of failure of anticoagulation or progression of PVT despite anticoagulation, or as additional endovascular treatment in patients treated with TIPS, often with good effectiveness and limited adverse events. However, the paucity and heterogeneity of the studies and the lack of large or randomized studies still preclude firm recommendations on such issue. Nonetheless, the high mortality rate of patients with acute, symptomatic PVT failing anticoagulation calls for early, aggressive rescue treatments. Thrombolysis, provided the absence of major contraindications, may be lifesaving in selected patients. A proper timing of thrombolysis, less than 30 days and, if possible, less than 14 days from the acute onset of PVT appears to achieve good results. Decision to carry out thrombolysis must take into account both the occurrence of major contraindications and the current critical clinical setting. In the next future, large high-quality multicentre studies will hopefully be able to establish more firm indications and preferable techniques.
